# Sulfisoxazole Elicits Robust Antitumour Immune Response Along with Immune Checkpoint Therapy by Inhibiting Exosomal PD‐L1

**DOI:** 10.1002/advs.202103245

**Published:** 2021-12-20

**Authors:** Jung Min Shin, Chan‐Hyeong Lee, Soyoung Son, Chan Ho Kim, Jae Ah Lee, Hyewon Ko, Sol Shin, Seok Ho Song, Seong‐Sik Park, Ju‐Hyun Bae, Ju‐Mi Park, Eun‐Ji Choe, Moon‐Chang Baek, Jae Hyung Park

**Affiliations:** ^1^ School of Chemical Engineering College of Engineering Sungkyunkwan University 2066 Seobu‐ro, Jangan‐gu Suwon 16419 Republic of Korea; ^2^ Department of Genetic Resources National Marine Biodiversity Institute of Korea (MABIK) 75 Jangsan‐ro 101‐gil, Janghang‐eup Seocheon 33662 Republic of Korea; ^3^ Department of Molecular Medicine CMRI Exosome Convergence Research Center (ECRC) School of Medicine Kyungpook National University Daegu 41944 Republic of Korea; ^4^ Department of Health Sciences and Technology SAIHST Sungkyunkwan University 2066 Seobu‐ro, Jangan‐gu Suwon 16419 Republic of Korea; ^5^ Bionanotechnology Research Center Korea Research Institute of Bioscience & Biotechnology 125 Gwahak‐ro, Yuseong‐gu Daejeon 34141 Republic of Korea; ^6^ Biomedical Institute for Convergence at SKKU (BICS) Sungkyunkwan University 2066 Seobu‐ro, Jangan‐gu Suwon 16419 Republic of Korea

**Keywords:** combination therapy, exosomal PD‐L1, exosome, immune checkpoint therapy, immune escape, tumor microenvironment

## Abstract

Despite their potent antitumor activity, clinical application of immune checkpoint inhibitors has been significantly limited by their poor response rates (<30%) in cancer patients, primarily due to immunosuppressive tumor microenvironments. As a representative immune escape mechanism, cancer‐derived exosomes have recently been demonstrated to exhaust CD8^+^ cytotoxic T cells. Here, it is reported that sulfisoxazole, a sulfonamide antibacterial, significantly decreases the exosomal PD‐L1 level in blood when orally administered to the tumor‐bearing mice. Consequently, sulfisoxazole effectively reinvigorates exhausted T cells, thereby eliciting robust antitumor effects in combination with anti‐PD‐1 antibody. Overall, sulfisoxazole regulates immunosuppression through the inhibition of exosomal PD‐L1, implying its potential to improve the response rate of anti‐PD‐1 antibodies.

## Graphical abstract

Biogenesis of cancer‐derived exosomal PD‐L1, responsible for the low response rates of the immune checkpoint inhibitors, is remarkably inhibited by sulfisoxazole as an endothelin receptor A‐selective antagonist. When orally administered to the tumor‐bearing mice, sulfisoxazole reinvigorates exhausted T cells, thereby eliciting potent antitumor effects in combination with an anti‐PD‐1 antibody.

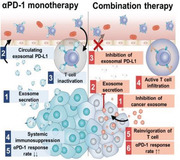

## 1 Introduction

Immunotherapy, one of the most potent strategies for the treatment of cancer, provides excellent clinical benefits by modulating the immune system of the body to boost innate antitumor activity.^[^ ^1^ ^]^ Thus, cancer immunotherapies [e.g., cancer vaccination, immune checkpoint blockade, or chimeric antigen receptor (CAR)‐T cell therapy] have recently emerged as promising alternatives to conventional treatments (e.g., chemotherapy, surgery, and radiation) and candidates for combinatorial approaches.^[^ ^2^ ^]^ In particular, immune checkpoint inhibitors are considered as one of the most promising therapeutic options because they have been shown to cause significant tumor remission in the clinic in various cancer types such as melanoma, breast cancer, and lung cancer.^[^ ^3^ ^]^ Unlike autologous dendritic cell‐based vaccines and CAR‐T cells, immune checkpoint inhibitors can be mass‐produced and are available to all cancer patients.

In immune checkpoint therapy (ICT), cancer patients are treated with monoclonal antibodies against specific immune checkpoint molecules, such as PD‐L1, PD‐1, and CTLA‐4.^[^ ^4^ ^]^ Once the negative regulation by the immune checkpoint is inhibited, the function of cytotoxic T cells is reinvigorated, which then eliminates cancer cells leading to the remission of tumors.^[^ ^5^ ^]^ However, a considerable proportion of cancer patients (>70%) do not respond to immune checkpoint inhibitors because cancer cells often create immunosuppressive microenvironments as part of their immune escape mechanisms.^[^ ^6^ ^]^ To address this limitation of ICT, it is necessary to develop a novel therapeutic approach that can boost cytotoxic T cells and neutralize the immune escape mechanisms of cancer.

**Figure 1:**
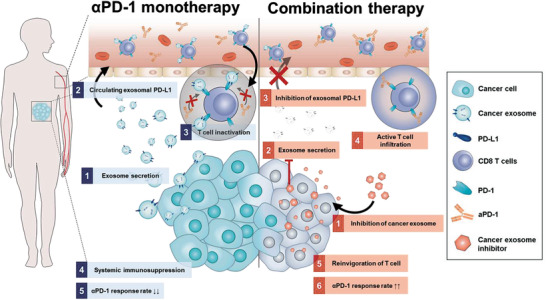
Schematic illustration depicting the mechanism of action of combination therapy using sulfisoxazole (SFX) and anti‐PD‐1 (*α*PD‐1). SFX, an FDA‐approved ETA antagonist, inhibits cancer exosome biogenesis and synergistically enhances the antitumor effect of *α*PD‐1. 1) Tumors actively secrete exosome with PD‐L1 (exosomal PD‐L1), which inhibits T cell activation as an immune escape mechanism in *α*PD‐1 monotherapy. 2) SFX inhibits exosome biogenesis in tumors, leading to enhanced antitumor efficacy of *α*PD‐1.

Exosomes (EXOs) (50–200 nm in diameter) produced by most eukaryotic cells play a critical role in intercellular communication by interacting with the receptors or delivering bioactive cargos into the recipient cells.^[^ ^7^ ^]^ Recently, it has been demonstrated that cancer EXOs are primarily responsible for immune escape mechanisms and subsequent low response (<30%) to ICT.^[^ ^8^ ^]^ To exhaust of the CD8^+^ cytotoxic T cells, tumor cells not only express PD‐L1 on their surface, but also secrete exosomal PD‐L1 through the fusion of multivesicular bodies with the plasma membrane.^[^ ^8^, ^9^ ^]^ In particular, excessive amount of exosomal PD‐L1 in the blood inhibits anti‐PD‐1 antibody (*α*PD‐1) by reducing its potential to preserve the bioactivity of CD8^+^ cytotoxic T cells. In fact, genetic reduction of PD‐L1 (+) EXO amounts in Rab27a^−^ or nsMase‐knockout tumor cells suppressed tumor growth in an immune‐dependent fashion.^[^ ^9a^ ^]^ To overcome this limitation of ICT induced by cancer EXOs, it is necessary to design strategies to control EXO secretion in cancers. In a previous study, we reported that sulfisoxazole (SFX) suppresses tumor growth and metastasis by inhibiting EXO secretion by targeting endothelin receptor A (ETA).^[^ ^10^ ^]^ This finding implied that the inhibition of tumor‐derived EXOs by SFX has the potential to suppress immune evasion by tumor‐derived exosomal PD‐L1. Therefore, we hypothesized that SFX, which prevents the biogenesis of cancer EXOs, could markedly increase the response rate to ICT by switching nonresponders to responders (**Figure** **1**). In this study, we found that SFX significantly decreased exosomal PD‐L1 levels in blood and activated CD8^+^ cytotoxic T cells when combined with *α*PD‐1 in animal models. Our findings imply that SFX modulates the immunosuppressive tumor microenvironment (TME) by inhibiting of exosomal PD‐L1 and can thus be used as a potential agent to increase the reactivity of *α*PD‐1.

## 2 Results and Discussion

### 2.1 SFX Inhibits Cancer EXO Secretion and Suppresses Exosomal PD‐L1

**Figure 2:**
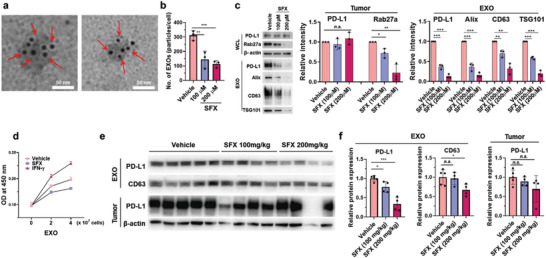
Sulfisoxazole (SFX) inhibits cancer exosome (EXO) biogenesis and suppresses exosomal PD‐L1. a) TEM images of MDA‐MB231‐derived EXOs immunogold‐labeled with *α*PD‐L1 antibodies. Arrowheads indicate 5 nm gold particles. Scale bar, 50 nm. b) Quantification of secreted EXOs in the presence of different concentrations of SFX (*n* = 3). c) Immunoblot for the indicated proteins in cell lysates and EXOs from MDA‐MB231 cells. Exosomal proteins, obtained from equal number of cells (1 × 10^7^), were loaded per lane (*n* = 3). d) PD‐1 binding to MDA‐MB231‐derived EXOs, obtained in the presence of SFX (200 × 10^−6^ m) or IFN‐*γ* (10 ng mL^−1^). e,f) Immunoblot of human CD63 and PD‐L1 in circulating EXOs and tumor lysates from MDA‐MB231 xenograft models. *β*‐actin was used as the loading control for tumor lysates. (Vehicle; *n* = 5 and SFX; *n* = 4). Significance was determined using an unpaired two‐tailed Student's *t*‐test. ***p < 0.001, **p < 0.01, and *p < 0.05. Error bar, standard deviation (SD).

To determine whether SFX reduces exosomal PD‐L1 through the regulation of EXO secretion, we quantified MDA‐MB231‐derived cancer EXOs following treatment with different concentrations of SFX both in vitro and in vivo (**Figure** **2**). As expected, cancer EXOs contained a large amount of PD‐L1 on their surface, and SFX efficiently inhibited EXO biogenesis in a dose‐dependent manner without affecting the PD‐L1 level on the cellular surface (Figure 2a–c). Interestingly, SFX downregulated the expression of Rab27a, a key enzyme responsible for EXO biogenesis, resulting in a significant reduction in exosomal PD‐L1 (Figure 2c). To assess the binding of exosomal PD‐L1 to PD‐1 protein, cancer EXOs were obtained from MDA‐MB231 cells in the presence of interferon gamma (IFN‐*γ*) or SFX (Figure 2d). IFN‐*γ*, a potent PD‐L1 inducer, increased the exosomal binding capacity to PD‐1, which may be due to the elevated PD‐L1 level on the exosomal surface. Conversely, owing to its inhibitory effect on exosomal secretion, SFX significantly reduced the exosomal binding capacity to PD‐1. These results suggest that SFX has the potential to suppress the binding of PD‐1 expressed in lymphocytes to tumor‐derived exosomal PD‐L1 by inhibiting exosomal secretion. Next, to investigate whether SFX reduces circulating exosomal PD‐L1, we determined the amount of exosomal PD‐L1 in plasma after oral administration of SFX to MDA‐MB231 xenograft models. Tumor‐derived EXOs and exosomal PD‐L1 were observed using human anti‐CD63 and anti‐PD‐L1 antibodies, respectively (Figure 2e,f).^[^ ^11^ ^]^ There was no significant change in the PD‐L1 level following SFX treatment in the tumor lysate, indicating that SFX did not affect PD‐L1 expression in tumor cells. These results suggest that SFX effectively reduces EXO and exosomal PD‐L1 both in vitro and in vivo. It is noteworthy that SFX effectively reduced tumor‐derived EXOs as well as circulating exosomal PD‐L1 associated with immunosuppressive TME.

### 2.2 SFX Reinvigorates T Cell Activity by Inhibiting Cancer EXO Secretion

**Figure 3:**
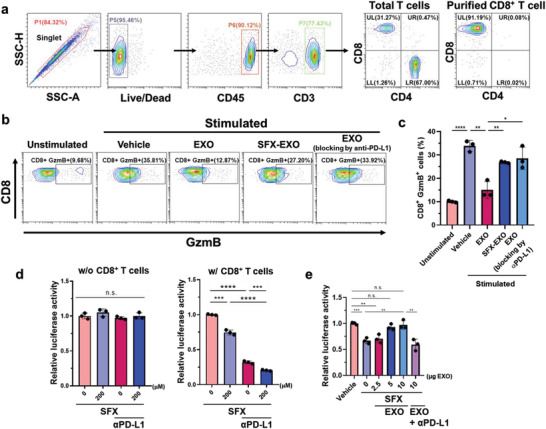
Sulfisoxazole (SFX) reinvigorates T cell activity by inhibiting of cancer exosome (EXO) biogenesis. a) Gating strategy used to identify the purified CD8^+^ T cells from human peripheral blood mononuclear cells (PBMCs). b) Representative contour plots of granzyme B (GzmB) in human CD8^+^ T cells with the indicated treatments. EXOs obtained from equal number of cells (2 × 10^7^ cells) were used. c) Quantification of CD8^+^GzmB^+^ cells in CD8^+^ T cells (*n* = 3). d) CD8^+^ T cells from human PBMCs were co‐cultured with MDA‐MB231‐luc (+) cells and then treated with or without SFX and *α*PD‐L1. Tumor cell death was evaluated by luciferase activity using an Alpha microplate reader (*n* = 3). e) CD8^+^ T cell‐mediated tumor killing assay in MDA‐MB231‐luc (+) cells incubated with SFX, exosomes, or PD‐L1‐ blocked EXOs by *α*PD‐L1 (*n* = 3). Significance was determined using an unpaired two‐tailed Student's *t*‐test. ***p < 0.001, **p < 0.01, and *p < 0.05. Error bar, standard deviation (SD).

EXO‐mediated immunosuppression is primarily based on the interaction between PD‐L1 on tumor‐derived EXOs and PD‐1 on CD8^+^ cytotoxic T cells.^[^ ^9b^ ^]^ Therefore, we investigated the effect of SFX on the activity of CD8^+^ cytotoxic T cells isolated from human peripheral blood mononuclear cells (PBMCs) (**Figure** **3**a). Briefly, tumor‐derived EXOs, obtained in the presence or absence of SFX, were treated with CD8^+^ cytotoxic T cells to observe changes in their bioactivity. The levels of granzyme B (GzmB) in CD8^+^ T cells was significantly reduced following treatment with MDA‐MB23‐derived EXOs, implying that exosomal PD‐L1 attenuated the bioactivity of CD8^+^ T cells (Figure 3b,c). Notably, compared to EXOs derived from vehicle‐treated cells, EXOs isolated from SFX‐treated cells showed a lower inhibitory effect on CD8^+^ T cells. In addition, treatment with EXOs pre‐incubated with anti‐PD‐L1 antibodies, nearly abolished their inhibitory effect on CD8^+^ T cells. Next, we examined whether SFX affected the killing function of CD8^+^ T cells. Tumor cell death was observed in MDA‐MB231‐luciferase cells in the presence of SFX with and without CD8^+^ T cells (Figure 3d). In the absence of CD8^+^ T cells, no significant difference in cancer cell viability was observed, regardless of SFX or *α*PD‐L1. However, cell viability was significantly reduced at higher concentrations of SFX, which may be due to the inhibition of the biogenesis of cancer EXOs. Interestingly, combined administration of *α*PD‐L1and SFX showed a synergistic effect without drug cytotoxicity. These results suggest that SFX maintains the bioactivity of CD8^+^ T cells by inhibiting secretion of exosomal PD‐L1.

### 2.3 SFX Synergistically Enhances the Antitumor Effect of Immune Checkpoint Inhibitors

To demonstrate the therapeutic efficacy of SFX in combination with *α*PD‐1, CT26 colon cancer cells were used to generate a tumor‐bearing mouse model (Figure S1, Supporting Information). Murine CT26 cells were chosen for additional in vivo experiments because they overexpress ETA (Figure S1, Supporting Information) and are available for cancer immunotherapy. Like MDA‐MB231 cells, CT26 cells showed no significant change in PD‐L1 expression following treatment with SFX. In addition, SFX efficiently inhibited the biogenesis of cancer‐derived EXOs and exosomal PD‐L1 from CT26 cells in a dose‐dependent manner by downregulating the expression of Rab27a (Figure S2, Supporting Information). These results indicate that the CT26 cell line is suitable for investigating the in vivo antitumor efficacy by regulation of exosomal PD‐L1 in the immunocompetent mice model.

**Figure 4:**
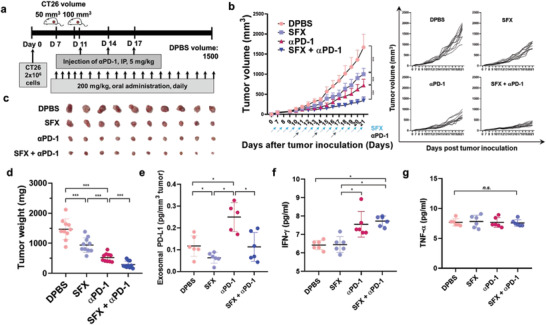
Sulfisoxazole (SFX) synergistically enhances the antitumor effect of an immune checkpoint inhibitor. a) Schematic illustration of the therapeutic schedule for CT26 tumor‐bearing mice. b) Antitumor effects of SFX, *α*PD‐1, and SFX + *α*PD‐1 (*n* = 10). c) Photographs of the tumors harvested on day 21 (*n* = 10). d) Tumor weight after treatment (*n* = 10). e) Quantification of exosomal PD‐L1 in mouse plasma after therapeutic regime (*n* = 10). f, g) Cytokine levels in plasma were quantified using ELISA (*n* = 6). Significance was determined using an ANOVA with Tukey correction. ***p < 0.001, **p < 0.01, and *p < 0.05. Error bar, standard deviation (SD).

To evaluate the effect of SFX on the antitumor response to *α*PD‐1, CT26 tumor‐bearing mice received the following treatments (**Figure** **4**a): Dulbecco's phosphate buffered saline (DPBS), SFX, *α*PD‐1, and SFX + *α*PD‐1 (SFX dose: 200 mg kg^−1^, *α*PD‐1 dose: 5 mg kg^−1^). As shown in Figure 4b, tumor growth was effectively retarded by SFX, *α*PD‐1, and SFX + *α*PD‐1. Notably, compared to *α*PD‐1, SFX + *α*PD‐1 significantly reduced the tumor growth rate, indicating a synergistic anticancer effect by SFX‐mediated inhibition of exosomal PD‐L1. These results imply that SFX boosts the in vivo antitumor effect of *α*PD‐1 in the immunocompetent mice. The data for tumor weights and images of the excised tumors were consistent with the results of the tumor volumes in the various groups (Figure 4c,d).

After evaluation of the antitumor efficacy, exosomal PD‐L1 in the mouse plasma in each group was evaluated using ELISA. As the basal exosomal PD‐L1 level in wild‐type mice are negligible compared to those in tumor‐bearing mice, tumor‐derived EXOs would primarily reflect the exosomal PD‐L1 levels in tumor‐bearing mice (Figure S3, Supporting Information). As shown in Figure 4e, SFX alone significantly decreased an exosomal PD‐L1 levels in the plasma, primarily due to inhibition of exosomal secretion. In contrast, *α*PD‐1 alone markedly increased the exosomal PD‐L1 level in the plasma, which may be due to the overexpression of PD‐L1 in cancer cells stimulated by IFN‐*γ*.^[^ ^12^ ^]^ This enhanced exosomal PD‐L1 level, causing CD8^+^ cytotoxic T cell exhaustion, is responsible for the limited antitumor efficacy of *α*PD‐1 (Figure 4b). Interestingly, when *α*PD‐1 was combined with SFX, exosomal PD‐L1 was restored to the basal level, comparable to that in the DPBS group. In addition, SFX + *α*PD‐1 increased the secretion of IFN‐*γ*, a representative inflammatory cytokine that is secreted by activated immune cells and plays an important role in antitumor immunity (Figure 4f).^[^ ^13^ ^]^ Therefore, *α*PD‐1 may elicit a strong antitumor immune response by minimizing exosomal PD‐L1‐mediated CD8^+^ cytotoxic T cell exhaustion in the presence of SFX. When considering the effect of SFX on the inhibition of exosomal secretion, *α*PD‐L1 is another promising candidate for combination therapy. As shown in Figure S4 (Supporting Information), the SFX + *α*PD‐L1 group exhibited much higher antitumor efficacy than the SFX‐ or *α*PD‐L1‐treated groups. Therefore, *α*PD‐L1 is expected to induce an enhanced antitumor immune response by avoiding exosomal PD‐L1‐mediated neutralization in the presence of SFX. Overall, SFX markedly enhanced the antitumor efficacy of immune checkpoint inhibitors by inhibiting exosomal secretion.

### 2.4 SFX Potentiates Antitumor Immune Response by Suppressing Exosomal PD‐L1

**Figure 5:**
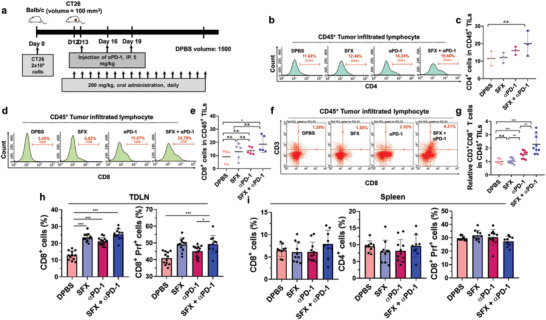
Combination of sulfisoxazole (SFX) and *α*PD‐1 elicits adaptive immunity against tumor. a) Schematic illustration of the therapeutic schedule for CT26 tumor‐bearing mice. b) Representative histogram of CD45^+^CD4^+^ cells in tumor microenvironment (TME). c) Quantification of CD45^+^CD4^+^ cells in the TME (*n* = 3). d) Representative histogram of CD45^+^CD8^+^ cells in TME. e) Quantification of CD45^+^CD4^+^ cells in TME (*n* = 5). f) Representative dot plot of CD45^+^CD3^+^CD8^+^ cytotoxic T cells in the TME. g) Quantification of CD45^+^CD3^+^CD8^+^ cytotoxic T cells in the TME (*n* = 9). h) Quantification of perforin^+^ T cells in CD8^+^ T cells from tumor‐draining lymph nodes (DPBS; *n* = 12, SFX; *n* = 12, *α*PD‐1; *n* = 14 and SFX + *α*PD‐1; *n* = 11). i) Quantification of CD4^+^ T cells, CD8^+^ T cells, and perforin^+^ T cells in CD8^+^ T cells from spleen (DPBS; *n* = 9, SFX; *n* = 10, *α*PD‐1; *n* = 11 and SFX+ *α*PD‐1; *n* = 9). Significance was determined using an ANOVA with Tukey correction or an unpaired two‐tailed Student's *t*‐test. ***p < 0.001, **p < 0.01, and *p < 0.05. Error bar, standard deviation (SD).

Given that SFX reduced exosomal PD‐L1 and elevated IFN‐*γ*, we sought to evaluate the generation of antitumor immunity by analyzing the immune cells in the tumor microenvironment, tumor‐draining lymph node, and spleen. To further evaluate the depletion effect of exosomal PD‐L1 in TME, the therapeutic schedule was slightly modified, allowing for obtaining sufficient immune cells in tumor tissue (**Figure** **5**a). As expected, the antitumor efficacy increased in the order of DPBS < SFX < *α*PD‐1 < SFX + *α*PD‐1 (Figure S5, Supporting Information). As evidenced by the results in Figure 4, inhibition of exosomal secretion may have greater therapeutic effect on smaller tumors at early stages.

Tumor‐infiltrating lymphocytes (TILs), which play a critical role in antitumor immune response, trigger the cancer immunity cycle by provoking apoptotic cell death and producing cancer‐associated antigens. In this study, CD45^+^ TILs were isolated from the excised tumors to investigate the effect of SFX on the antitumor response to *α*PD‐1. In the SFX + *α*PD‐1 group, the number of CD4^+^ cells in the TME was comparable to that in the other groups, implying that CD4^+^ T cells were not a major subset mediating the synergistic effect of SFX and *α*PD‐1 (Figure 5b,c and Figure S6, Supporting Information). Compared to DPBS, SFX + *α*PD‐1 significantly increased CD8^+^ TILs in the TME (Figure 5d,e and Figure S6, Supporting Information). Further, *α*PD‐1 increased the population of CD3^+^ CD8^+^ cytotoxic T cells in the TME due to its specific binding to PD‐1 on CD8^+^ cytotoxic T cells (Figure 5f,g). Interestingly, compared to *α*PD‐1 alone, SFX + *α*PD‐1 significantly increased the levels of CD3^+^CD8^+^ TILs in the TME, implying that inhibition of exosomal PD‐L1 enhances the bioactivity of *α*PD‐1 to induce a strong anticancer immune response. CD4/CD8 TIL ratios of less than 1 in the TME are associated with clinically poor prognosis in cancer patients.^[^ ^14^ ^]^ The ratios for mice treated with DPBS and SFX were below 1, whereas those for *α*PD‐1 or SFX + *α*PD‐1 were above 1 (Figure S7, Supporting Information). SFX, *α*PD‐1, and SFX + *α*PD‐1 increased the number of CD8^+^ T cells and CD8^+^ perforin^+^ T cells in the draining lymph nodes, suggesting the induction of adaptive immunity (Figure 5h). In contrast, immunophenotyping of the spleen did not reveal any significant effect on the population or cytotoxic activity of CD8^+^ and CD4^+^ T cells (Figure 5i). Overall, SFX‐mediated inhibition of tumor‐derived exosomal PD‐L1 enhanced the CD8^+^ T cell‐mediated antitumor immune response. These results suggest that SFX in combination with *α*PD‐1 elicits a robust antitumor immune response.

## 3 Conclusion

Although overexpressed PD‐L1 on cancer cell by IFN‐*γ* significantly contributes to the immunosuppressive TME, *α*PD‐1 antibody still binds to PD‐1 on circulating CD8^+^ cytotoxic T cells, leading to its effective antitumor efficacy. In contrast, exosomal PD‐L1 competitively binds to circulating CD8^+^ cytotoxic T cells in the blood, exhausting the CD8^+^ cytotoxic T cells. In the presence of exosomal PD‐L1, *α*PD‐1 antibody is no longer bound to CD8^+^ cytotoxic T cells, resulting in diminished therapeutic efficacy. Therefore, inhibition of exosomal PD‐L1 is an excellent option to overcome the immune escape of cancer in ICT.

In this study, we devised an unprecedented therapeutic strategy to reduce exosomal PD‐L1 via inhibitory regulation of exosome biogenesis, thereby enhancing *α*PD‐1 antibody‐based immune response. To validate this strategy, we evaluated the effect of SFX in inhibiting cancer EXOs both in vitro and in vivo. SFX significantly suppressed exosomal secretion in proportion to the concentration, thus reducing the level of exosomal PD‐L1 in blood. Therefore, T cell exhaustion by exosomal PD‐L1 was efficiently prevented by SFX. Consequently, when combined with *α*PD‐1 antibody, SFX dramatically improved antitumor response by increasing CD8^+^ cytotoxic T cells in the TME.

## 4 Experimental Section

### Materials

*α*PD‐1 and SFX were purchased from BioXCell (Lebanon, NH, USA) and Sigma‐Aldrich (St. Louis, MO, USA), respectively. The deionized water used in this study was purified using the AquaMax‐Ultra Water Purification System (Anyang, Republic of Korea). All other chemicals were used as received without further purification.

### Cell Lines and Cell Culture

Human breast cancer MDA‐MB231 and murine colon cancer CT26 cells were obtained from the American Type Culture Collection (Manassas, VA, USA). MDA‐MB231 cells were cultured in Dulbecco's modified Eagle's medium (DMEM) supplemented with 10% fetal bovine serum (FBS) and 1% antibiotic/antimycotic solution. CT26 cells were cultured in RPMI supplemented with 10% FBS and 1% antibiotic/antimycotic solution. Human CD8^+^ T cells from PBMCs of healthy donors were cultured in RPMI supplemented with 20% FBS, 1% antibiotic/antimycotic solution, and 100 IU mL^−1^ human IL‐2.

### Isolation and Quantitation of EXO

EXOs were purified by differential centrifugation as described previously.^[^ ^10^, ^15^ ^]^ Briefly, cell supernatants were subjected to differential centrifugation at 300 × g/3 min, 2500 × g/15 min, and 10 000 × g/30 min. After filtration through a 0.22 µm filter, the supernatant was centrifuged at 120 000 × g for 90 min. The pellets were resuspended with phosphate‐buffered saline (PBS) and centrifuged at 120 000 × g/90 min again. The pellet (containing EXOs) was resuspended in PBS or RIPA lysis buffer for further analysis.

Mouse plasma EXOs were centrifuged at 2500 × g for 15 min and 10 000 × g for 30 min to remove cells and cell debris. The supernatant was then centrifuged at 120 000 × g for 90 min.

The EXO proteins were quantified using the Pierce BCA Protein Assay kit (Thermo Scientific, Waltham, MA, USA) after treatment with RIPA buffer [Cell Signaling Technology (CST), Danvers, MA, USA].

### Transmission Electron Microscopic Analysis

In order to obtain the TEM images for identification of exosomal surface proteins, gold nanoparticles (5–10 nm in diameter) have been extensively used.^[^ ^8^, ^16^ ^]^ In this study, exosomal PD‐L1 was observed using TEM with immunogold labeling, as reported previously.^[^ ^8a^ ^]^ Briefly, the purified EXOs were deposited onto pure carbon‐coated grids. For immunogold labeling, EXOs were treated with mouse *α*PD‐L1 (eBioscience, 14‐5983‐82, San Diego, CA, USA), followed by incubation with antimouse IgG‐conjugated 5 nm gold particles (Sigma‐Aldrich, G7527). After staining with 2% uranyl acetate, the grids were dried at 25 °C and visualized at 100 kV using a Hitachi HT‐7700 TEM (Tokyo, Japan).

### Nanoparticle Tracking Analysis (NTA)

The number of EXOs was measured using NTA as described in our previous study^[^ ^17^ ^]^. Suspensions containing EXOs from cell culture medium were analyzed using a NanoSight LM10 instrument (NanoSight, Wiltshire, UK). For this analysis, a monochromatic laser beam (405 nm) was applied to a dilute suspension of the EXOs. A video of 30 s duration was recorded at a rate of 30 frames s^−1^, and EXO movement was analyzed using NTA software (version 2.2; NanoSight). NTA postacquisition settings were optimized and kept constant between samples, and each video was analyzed to estimate the concentration.

### PD‐1 Binding Assay

PD‐1/PD‐L1 binding was evaluated as described previously.^[^ ^8a^ ^]^ In brief, to evaluate the binding of exosomal PD‐L1 to PD‐1, 96‐well ELISA plates were coated with 4 µg mL^−1^ human PD‐1 protein (BPS Bioscience, Cat# 71106, San Diego, CA, USA) overnight at 4 °C. The free binding sites were blocked with PBS containing 0.05% Tween 20 for 2 h at 25 °C. Then, 100 µL of the EXO samples were incubated overnight at 4 °C. After washing, 100 µL of 4 µg mL^−1^ biotin‐labeled PD‐L1 antibody (eBioscience, Cat# 13‐5983‐82) was added and the mixture was incubated for 2 h at 25 °C. Thereafter, horseradish peroxidase‐conjugated streptavidin was added, followed by incubation for 1 h at 25 °C. Plates were developed using 3,3,5,5‐tetramethylbenzidine‐containing peroxide. The reaction was stopped, and the absorbance was measured at 450 nm using an automated iMark (Bio‐Rad, Hercules, CA, USA).

### Isolation of CD8^+^ T Cells and Treatment with EXOs

Activity of CD8^+^ T cells was measured as described previously.^[^ ^8a^ ^]^ Purified EXOs were incubated with PD‐L1 antibodies (10 µg mL^−1^) in PBS, washed with 30 mL PBS, and pelleted by ultracentrifugation to remove the nonbound free antibodies. Human CD8^+^ T cells were purified from PBMCs using a human CD8^+^ T cell isolation kit (Miltenyi Biotec, Bergisch Gladbach, Germany). CD8^+^ T cells were activated with 2 µg mL^−1^ human CD3/CD28 antibody for 24 h and then incubated with MDA‐MB231‐derived EXOs with or without PD‐L1 blockage for 48 h in the presence of anti‐CD3/CD28 antibodies.

### CD8^+^ T Cell‐Mediated Tumor Killing Assay

MDA‐MB231‐luciferase cells (5000 cells per well) were plated in the 96‐well plate. Then, the cells were co‐cultured with activated human CD8^+^ T cells in the presence of SFX, *α*PD‐L1, or EXO for 48 h at an effector to target (E:T) ratio of 1:1. The plates were washed with PBS, and then 100 µL of 2 mg mL^−1^ luciferin was added to each well. The luciferase intensity in each well was immediately measured using an Alpha microplate reader (PerkinElmer, Waltham, MA, USA).

### Western Blot Assay

Cellular or EXO proteins were resolved by sodium dodecyl sulfate‐polyacrylamide gel electrophoresis, transferred onto nitrocellulose membranes, probed with the respective primary antibody, and incubated with a horseradish peroxidase‐linked secondary antibody. Images were visualized using enhanced chemiluminescence (ECL) detection reagents (#34580; Thermo Scientific) and quantified using ECL hyper‐film (AGFA; Morstel) and Fusion FX7 system (Vilber Lourmat, Eberhardzell, Germany). The following primary antibodies were used: anti‐CD63 (ab68418, 1:1000; Abcam, Cambridge, UK), anti‐Alix (ab56932, 1:1000; Abcam), anti‐CD9 (ab92726, 1:1000; Abcam), anti‐TSG101 (ab30871, 1:1000; Abcam), anti‐beta‐actin (4670, 1:20 000; CST), Rab27a (ab55667, 1:1000; Abcam), and PD‐L1 (#13684S; CST).

### Tumor Growth Inhibition

All animal procedures were approved by the Institutional Animal Care and Use Committees of the Sungkyunkwan University (SKKUIACUC2020‐05‐15‐2) and Kyungpook National University (KNUIACUC2020‐0016). CT26 cells (2 × 10^6^) were suspended in cold PBS and subcutaneously injected to establish CT26 tumor‐bearing mice. After 7 d, the mice were treated with DPBS, SFX, *α*PD‐1, or SFX + *α*PD‐1 (SFX: oral administration, *α*PD‐1: intraperitoneal administration), as described in Figure 4a (*n* = 10). The tumor volume was measured using calipers and calculated for each mouse using the following equation: *V* = 1/2*ab* ^2^, where *a* is the longest axis and *b* is the shortest axis. After the treatment schedule, the tumors were excised and weighed.

### Detection of PD‐L1 on EXOs

To isolate circulating human EXOs, MDA‐MB231 cells suspended in PBS were orthotopically injected into the left fat pad of 5‐week‐old female BALB/c nude mice. SFX (100–200 mg kg^−1^ d^−1^) was orally administered for 14 d. The mice were then euthanized and their plasma and tumors were extracted. To isolate circulating mouse EXOs, blood samples from the CT26 tumor‐bearing mice were collected at the end of the antitumor efficacy study. Plasma was isolated via centrifugation at 2000 × g for 20 min, and the cell‐free plasma was centrifuged at 16 500 × g for 45 min to remove microvesicles. EXOs were isolated using a total EXO isolation kit (Invitrogen, Cat# 4484450, Carlsbad, CA, USA). To assess PD‐L1 on EXOs isolated from mouse plasma, ELISA plates were coated with a monoclonal antibody against PD‐L1 (R&D Systems, Minneapolis, MN, USA) overnight at 25 °C. Free binding sites were blocked with blocking buffer for 2 h at 25 °C. After washing the plates with 0.05% Tween‐20 in PBS, EXOs were added to each well and incubated for 2 h at 25 °C. The EXO‐containing wells were then sequentially incubated with the biotinylated PD‐L1 antibody for 2 h and horseradish peroxidase‐conjugated streptavidin for 20 min at 25 °C. The plate was incubated for 20 min with a substrate solution composed of H_2_O_2_ and tetramethylbenzidine. After the addition of a stop solution containing 2N H_2_SO_4_ (R&D Systems, DY994), the plate was immediately read at 450 nm using an xMark microplate reader (Bio‐Rad).

### Flow Cytometric Analysis

Following the therapeutic schedule (as described in Figure 5a), the tumor tissue was removed and a single‐cell suspension was obtained using the gentleMACS Tumor Dissociation Kit (Miltenyi Biotec), according to the manufacturer's instructions. Then, CD45^+^ TILs were isolated using MACS beads (MicroBeads, Miltenyi Biotec). The isolated TILs were labeled with CD3 (PE labeled, Biolegend, San Diego, CA, USA), CD8 (FITC‐labeled, Biolegend), or CD4 (FITC‐labeled, Biolegend) antibodies. The cells were then analyzed using Guava easyCyte (EMD Millipore, Billerica, MA, USA).

### Immunofluorescence Staining

Paraffin‐embedded tissue sections were used for immunofluorescence staining. An antigen‐retrieval step was carried out in citrate buffer (pH 6.0), and the sections were blocked for 1 h with PBS containing 1% BSA. The antibodies were diluted with staining buffer (PBS with 1% BSA), according to the manufacturer's instructions. The sections were incubated with antibodies overnight at 4 °C and washed three times with PBS (pH 7.4). The following antibodies were used: anti‐CD8‐FITC (clone 53–6.7, Invitrogen), anti‐CD4‐FITC (clone GK1.5, Biolegend). After staining nuclei with DAPI Fluoromount‐G, images were obtained using a confocal microscope (LSM 510 META NLO, Heidelberg, Germany). Green and blue fluorescence intensities were analyzed by selecting a region of 0.1 mm^2^ for each sample and quantified using ZEN 3.2 software (ZEISS, Heidelberg, Germany).

### Statistical Analysis

Statistics were processed and analyzed by using GraphPad Prism 8.0 (GraphPad, USA). Statistical significance of the experimental results was assessed using one‐way analysis of variance (ANOVA) with Tukey correction or unpaired two‐tailed Student's *t*‐test. Error bars in the graphical data represent mean ± standard deviation. All in vitro experiments were performed in triplicate unless otherwise stated. A *p*‐value <0.05 was regarded as statistically significant (indicated with an asterisk (*) in the corresponding figures as follows: *p < 0.05, **p < 0.01, ***p < 0.001).

## Conflict of Interest

The authors declare no conflict of interest.

## Supporting information

Supporting Information

## Data Availability

The data that support the findings of this study are available from the corresponding author upon reasonable request.

## References

[1] a) Y. Yang , J. Clin. Invest. 2015, 125, 3335; 26325031 10.1172/JCI83871 PMC4588312

[2] a) E. Blass , P. A. Ott , Nat. Rev. Clin. Oncol. 2021, 10.1038/s41571-020-00460-21; PMC7816749 33473220

[3] a) C. Robert , J. Schachter , G. V. Long , A. Arance , J. J. Grob , L. Mortier , A. Daud , M. S. Carlino , C. McNeil , M. Lotem , J. Larkin , P. Lorigan , B. Neyns , C. U. Blank , O. Hamid , C. Mateus , R. Shapira‐Frommer , M. Kosh , H. Zhou , N. Ibrahim , S. Ebbinghaus , A. Ribas , N. Engl. J. Med. 2015, 372, 2521; 25891173 10.1056/NEJMoa1503093

[4] P. Sharma , J. P. Allison , Cell 2015, 161, 205. 25860605 10.1016/j.cell.2015.03.030 PMC5905674

[5] A. D. Waldman , J. M. Fritz , M. J. Lenardo , Nat. Rev. Immunol. 2020, 20, 651. 32433532 10.1038/s41577-020-0306-5 PMC7238960

[6] W. J. Lesterhuis , A. Bosco , M. J. Millward , M. Small , A. K. Nowak , R. A. Lake , Nat. Rev. Drug Discovery 2017, 16, 264. 28057932 10.1038/nrd.2016.233

[7] a) S. Keller , J. Ridinger , A. K. Rupp , J. W. Janssen , P. Altevogt , J. Transl. Med. 2011, 9, 86; 21651777 10.1186/1479-5876-9-86 PMC3118335

[8] a) G. Chen , A. C. Huang , W. Zhang , G. Zhang , M. Wu , W. Xu , Z. Yu , J. Yang , B. Wang , H. Sun , H. Xia , Q. Man , W. Zhong , L. F. Antelo , B. Wu , X. Xiong , X. Liu , L. Guan , T. Li , S. Liu , R. Yang , Y. Lu , L. Dong , S. McGettigan , R. Somasundaram , R. Radhakrishnan , G. Mills , Y. Lu , J. Kim , Y. H. Chen , H. Dong , Y. Zhao , G. C. Karakousis , T. C. Mitchell , L. M. Schuchter , M. Herlyn , E. J. Wherry , X. Xu , W. Guo , Nature 2018, 560, 382; 30089911 10.1038/s41586-018-0392-8 PMC6095740

[9] a) M. Poggio , T. Hu , C. C. Pai , B. Chu , C. D. Belair , A. Chang , E. Montabana , U. E. Lang , Q. Fu , L. Fong , R. Blelloch , Cell 2019, 177, 414; 30951669 10.1016/j.cell.2019.02.016 PMC6499401

[10] E. J. Im , C. H. Lee , P. G. Moon , G. G. Rangaswamy , B. Lee , J. M. Lee , J. C. Lee , J. G. Jee , J. S. Bae , T. K. Kwon , K. W. Kang , M. S. Jeong , J. E. Lee , H. S. Jung , H. J. Ro , S. Jun , W. Kang , S. Y. Seo , Y. E. Cho , B. J. Song , M. C. Baek , Nat. Commun. 2019, 10, 1387. 30918259 10.1038/s41467-019-09387-4 PMC6437193

[11] N. Nishida‐Aoki , N. Tominaga , F. Takeshita , H. Sonoda , Y. Yoshioka , T. Ochiya , Mol. Ther. 2017, 25, 181. 28129113 10.1016/j.ymthe.2016.10.009 PMC5363297

[12] a) K. Abiko , N. Matsumura , J. Hamanishi , N. Horikawa , R. Murakami , K. Yamaguchi , Y. Yoshioka , T. Baba , I. Konishi , M. Mandai , Br. J. Cancer 2015, 112, 1501; 25867264 10.1038/bjc.2015.101 PMC4453666

[13] M. J. Smyth , Nat. Immunol. 2005, 6, 646. 15970935 10.1038/ni0705-646

[14] a) W. Shah , X. Yan , L. Jing , Y. Zhou , H. Chen , Y. Wang , Cell Mol. Immunol. 2011, 8, 59; 21200385 10.1038/cmi.2010.56 PMC4002991

[15] C. Théry , S. Amigorena , G. Raposo , A. Clayton , Curr. Protoc. Cell Biol. 2006, Chapter 3, Unit 3.22. 10.1002/0471143030.cb0322s30 18228490

[16] a) Y. Qiu , Y. Yang , R. Yang , C. Liu , J.‐M. Hsu , Z. Jiang , L. Sun , Y. Wei , C.‐W. Li , D. Yu , J. Zhang , M.‐C. Hung , Oncogene 2021, 40, 4992; 34172932 10.1038/s41388-021-01896-1 PMC8342306

[17] J. E. Lee , P. G. Moon , Y. E. Cho , Y. B. Kim , I. S. Kim , H. Park , M. C. Baek , J. Proteomics 2016, 131, 17. 26463135 10.1016/j.jprot.2015.10.005

