# Supplementary material for: Sulfisoxazole Elicits Robust Antitumour Immune Response Along with Immune Checkpoint Therapy by Inhibiting Exosomal PD‐L1
Source: Adv Sci (Weinh). 2021 Dec 20;9(5):2103245. doi: 10.1002/advs.202103245 (PMC8844465; doi:10.1002/advs.202103245)
Supplement: Supplementary file 1 — Supporting Information [file ADVS-9-2103245-s001.pdf]

WILEY-VCH

ADVANCED SCIENCE
Open Access

Supporting Information

for *Adv. Sci.*, DOI: 10.1002/advs.202103245

Sulfisoxazole elicits robust antitumour immune response along with immune checkpoint therapy by inhibiting exosomal PD-L1

Jung Min Shin, Chan-Hyeong Lee, Soyoung Son, Chan Ho Kim, Jae Ah Lee, Hyewon Ko, Sol Shin, Seok Ho Song, Seong-Sik Park, Ju-Hyun Bae, Ju-Mi Park, Eun-Ji Choe, Moon-Chang Baek * *and Jae Hyung Park* *

Supporting Information

Sulfisoxazole elicits robust antitumour immune response along with immune checkpoint therapy by inhibiting exosomal PD-L1

Jung Min Shin[1], Chan-Hyeong Lee[1], Soyoung Son, Chan Ho Kim, Jae Ah Lee, Hyewon Ko, Sol Shin, Seok Ho Song, Seong-Sik Park, Ju-Hyun Bae, Ju-Mi Park, Eun-Ji Choe, Moon-Chang Baek and Jae Hyung Park**

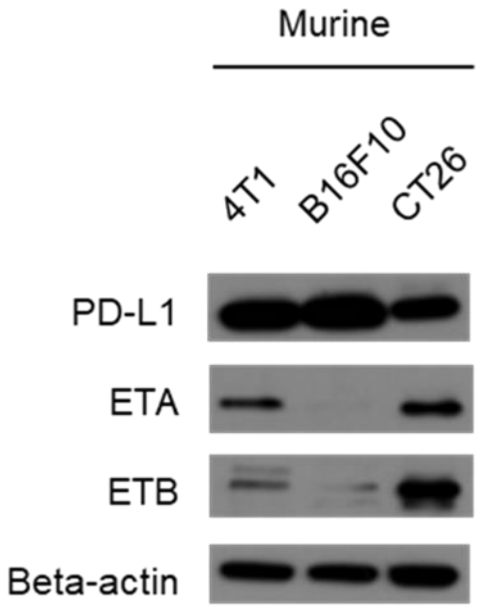

Figure S1. PD-L1, ETA, and ETB expression in murine cancer cell lines (4T1: breast cancer, B16F10: melanoma, CT26: colon cancer).

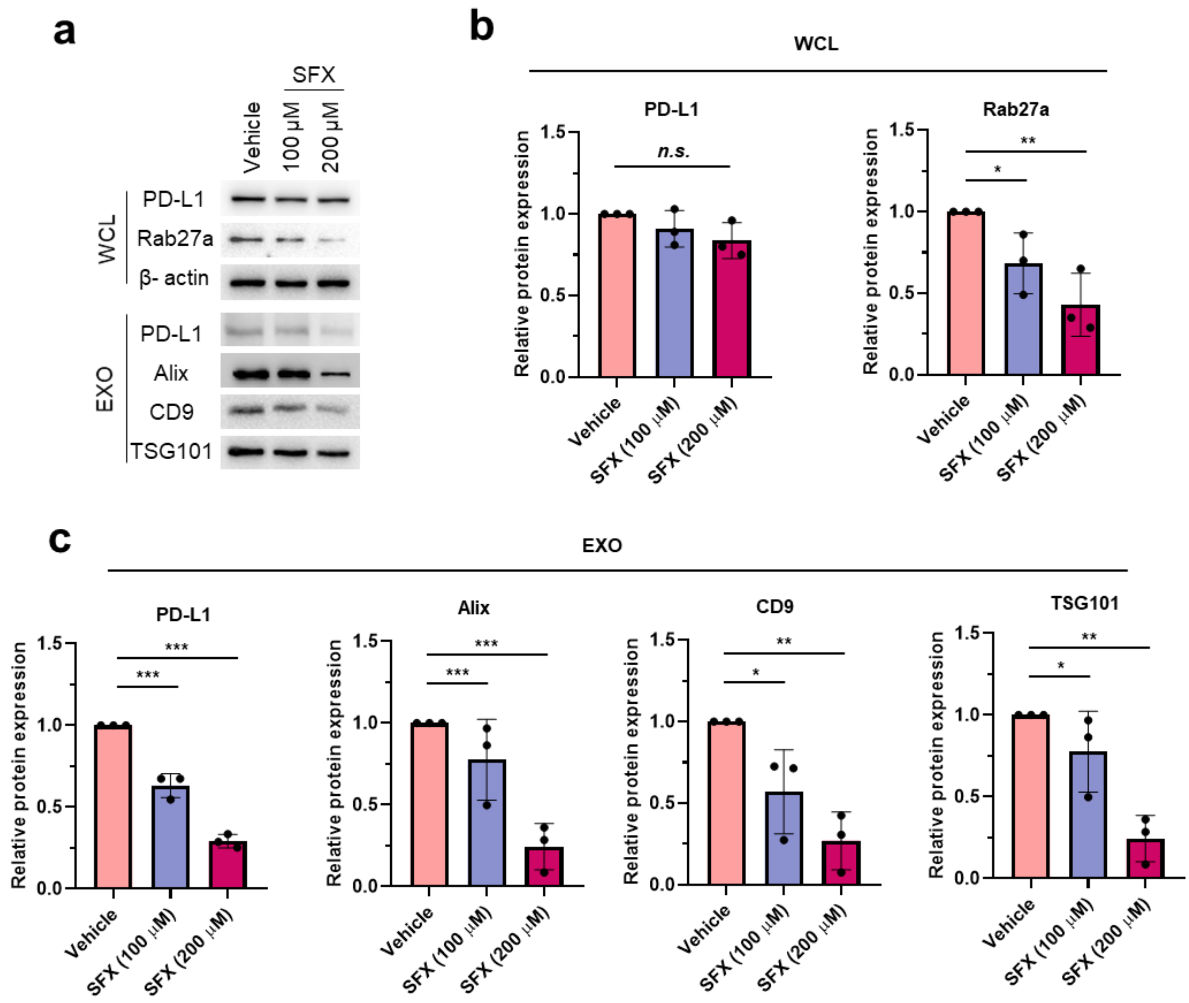

Figure S2. Sulfisoxazole (SFX) inhibits cancer EXO biogenesis and suppresses exosomal PD-L1 from CT26 murine colon cancer cell line. (a) Immunoblot for the indicated proteins in cell lysates and exosomes from CT26 cells treated with or without SFX. Beta-actin was used as loading controls for cell lysates. Exosomal proteins obtained from equal cell numbers (1 x 10^7) were loaded per lanes. (b) Quantification of PD-L1 and Rab27 in whole cell lysates (n = 3). (c) Quantification of exosomal proteins (n = 3). Significance was determined using an

unpaired two-tailed Student's *t*-test. ***$p<0.001$, **$p<0.01$ and *$p<0.05$. Error bar, standard deviation (SD).

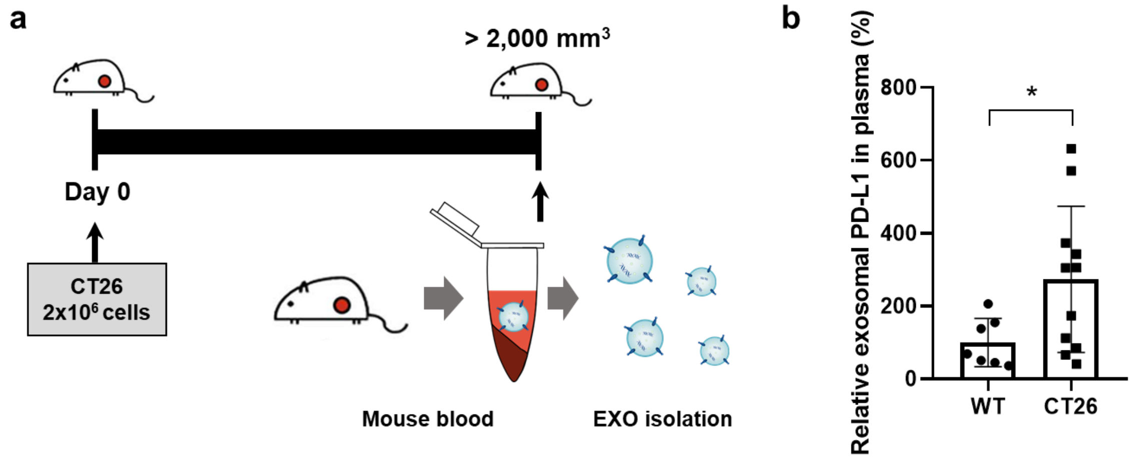

Figure S3. Quantification of exosomal PD-L1 in plasma from CT26 tumor-bearing mice. (a) Experimental regime of exosomal PD-L1 isolation. (b) Relative exosomal PD-L1 in plasma from WT and CT26 tumor-bearing mice (n = 7 and 11 for WT and CT26, respectively). Significance was determined using an unpaired two-tailed Student's *t*-test. ***$p<0.001$, **$p<0.01$ and *$p<0.05$. Error bar, standard deviation (SD).

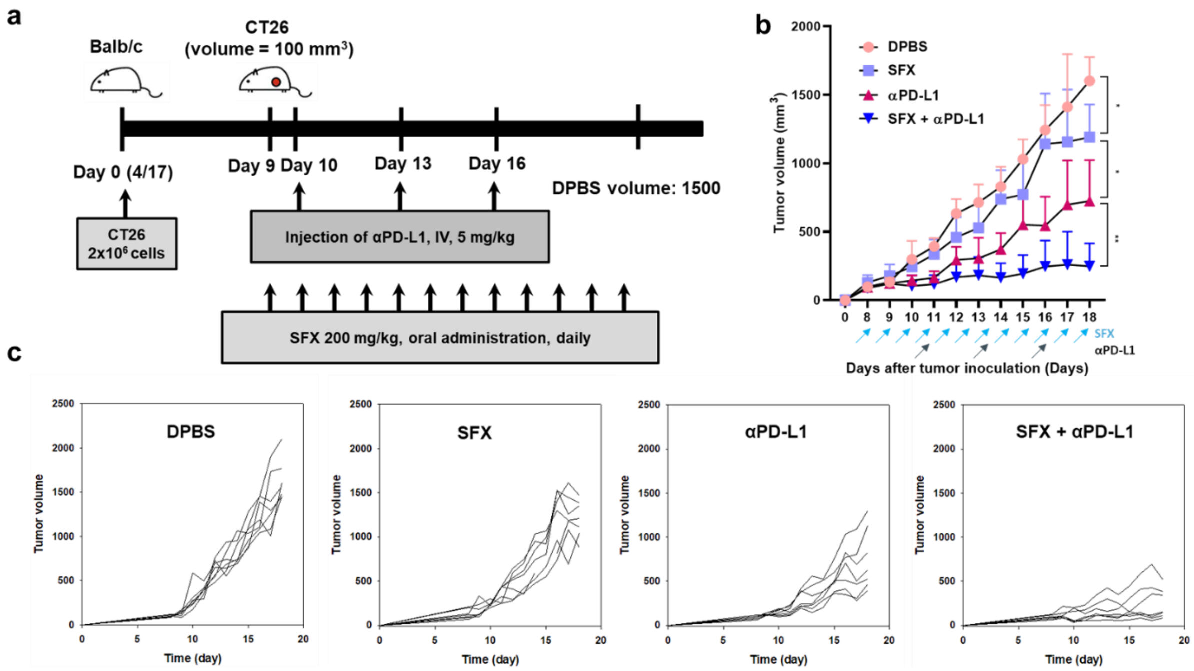

Figure S4. Antitumor efficacy of combinational treatment of SFX and αPD-L1 in CT26 tumor- bearing mice. (a) Experimental scheme for antitumor efficacy. (b) Average tumor volume (n = 7). (c) Individual tumor volume (n = 7). Significance was determined using an ANOVA with Tukey correction. ***p<0.001, **p<0.01 and *p<0.05. Error bar, standard deviation (SD).

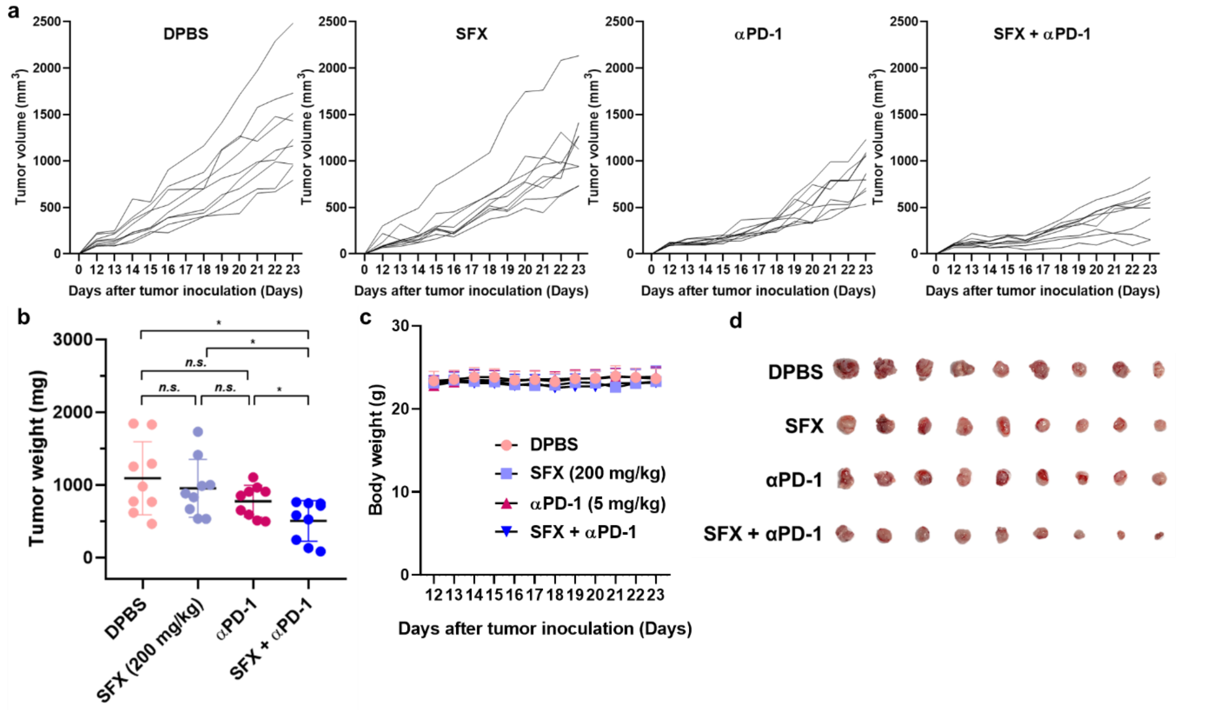

Figure S5. Antitumor efficacy of SFX, αPD-1, and SFX + αPD-1. (a) Individual tumor volume (n = 9). (b) Tumor weight after treatment (n = 9). (c) Changes in body weights (n = 9). (d) Photographs of the tumors harvested at 23 d (n = 9). Significance was determined using an ANOVA with Tukey correction. ***$p<0.001$, **$p<0.01$ and *$p<0.05$. Error bar, standard deviation (SD).

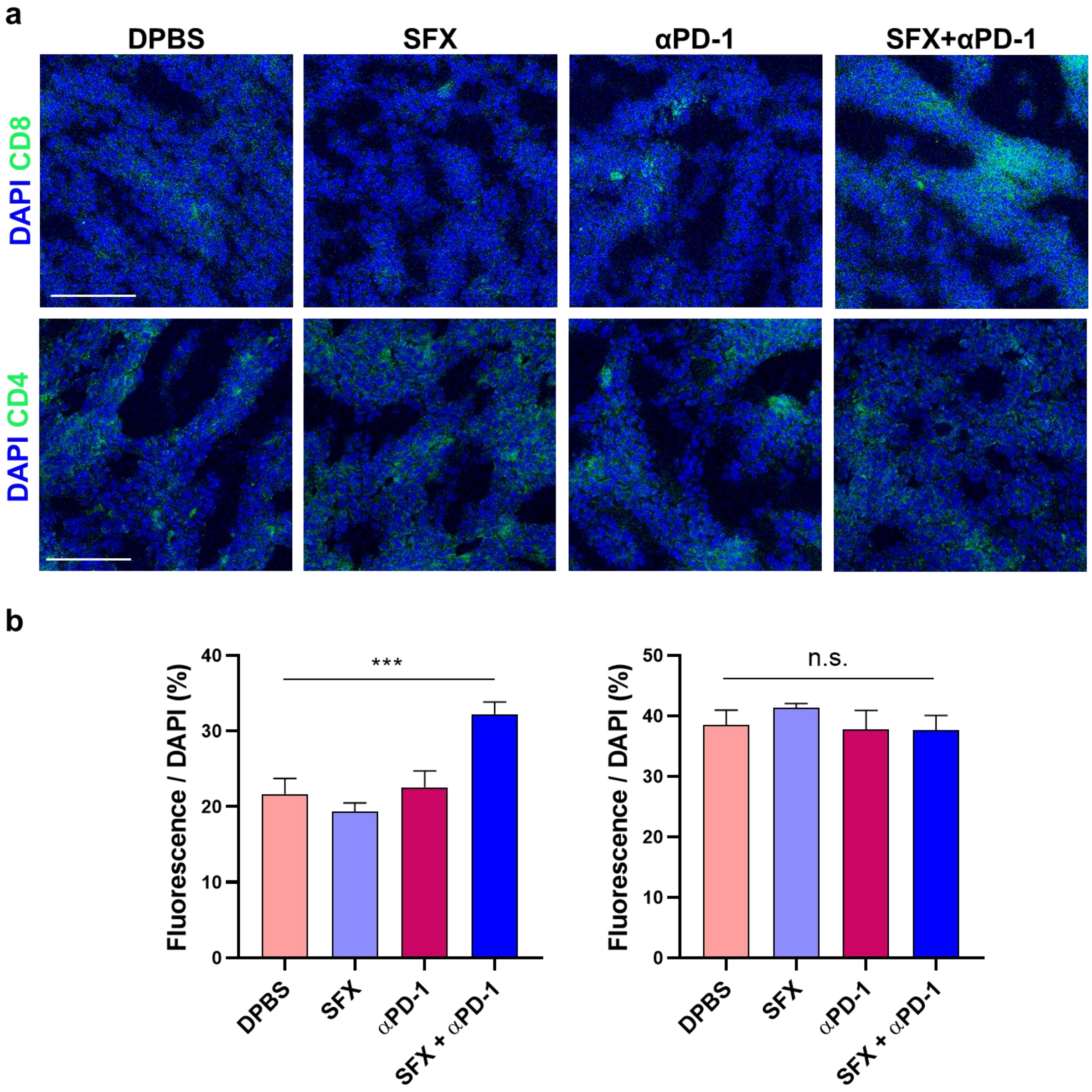

Figure S6. (a) Immunofluorescence microscopy images of $CD8^+$ (upper) and $CD4^+$ (lower) cells in the tumor microenvironment. Scale bars, 200 nm. Blue, cell nuclei. The images are representative of three independent experiments. (b) Relative value of fluorescence intensity to DAPI for CD8-FITC(left) and CD4-FITC(right), corresponding to data in **a** (n = 3)**.** Significance was determined using an ANOVA with Tukey correction. ***$p<0.001$, **$p<0.01$ and *$p<0.05$. Error bar, standard deviation (SD).

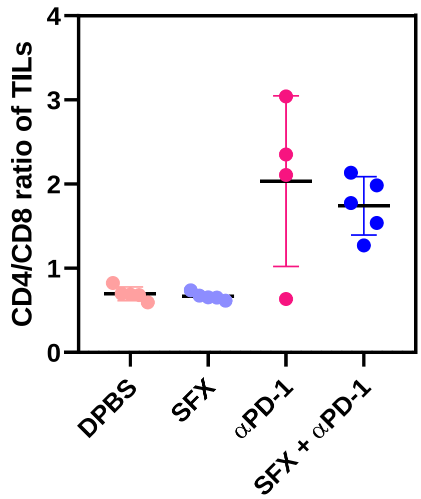

Figure S7. CD4/CD8 ratio of tumor-infiltrated lymphocytes in tumor microenvironment (DPBS ; *n* = 5, SFX ; *n* = 5, αPD-1 ; *n* = 4 and SFX+ αPD-1 ; *n* = 5).
